# A Novel Compound NSC745885 Exerts an Anti-Tumor Effect on Tongue Cancer SAS Cells In Vitro and In Vivo

**DOI:** 10.1371/journal.pone.0104703

**Published:** 2014-08-15

**Authors:** Yuan-Wu Chen, Hsu-Shan Huang, Yi-Shing Shieh, Kuo-Hsing Ma, Shing-Hwa Huang, Dueng-Yuan Hueng, Huey-Kang Sytwu, Gu-Jiun Lin

**Affiliations:** 1 School of Dentistry, National Defense Medical Center, Taipei, Taiwan, Republic of China; 2 Department of Oral and Maxillofacial Surgery, Tri-Service General Hospital, Taipei, Taiwan, Republic of China; 3 School of Pharmacy, National Defense Medical Center, Taipei, Taiwan, Republic of China; 4 Graduate Institute of Cancer Biology and Drug Discovery, College of Medical Science and Technology, Taipei Medical University, Taipei, Taiwan, Republic of China; 5 Department of Biology and Anatomy, National Defense Medical Center, Taipei, Taiwan, Republic of China; 6 Department of General Surgery, Tri-Service General Hospital, Taipei, Taiwan, Republic of China; 7 Department of Neurological Surgery, Tri-Service General Hospital, Taipei, Taiwan, Republic of China; 8 Department of Microbiology and Immunology, National Defense Medical Center, Taipei, Taiwan, Republic of China; Columbia University Medical Center, United States of America

## Abstract

**Objective:**

Oral squamous cell carcinoma (OSCC) is a prevalent cancer, especially in developing countries. Anthracyclines and their anthraquinone derivatives, such as doxorubicin, exhibit a cell growth inhibitory effect and have been used as anti-cancer drugs for many years. However, the cardiotoxicity of anthracycline antibiotics is a major concern in their clinical application. NSC745885 is a novel compound synthesized from 1,2-diaminoanthraquinone, which subsequently reacts with thionyl chloride and triethylamine. The present study aimed to investigate the anti-oral cancer potential and the safety of NSC745885.

**Methods:**

We investigated the anti-cancer potential of NSC745885 in oral squamous carcinoma cell lines and in an *in vivo* oral cancer xenograft mouse model. The expression of apoptotic related genes were evaluated by real-time RT-PCR and western bloting, and the *in vivo* assessment of apoptotic marker were measured by immunohistochemical staining. The anti-tumor efficiency and safety between doxorubicin and NSC745885 were also compared.

**Results:**

Our results demonstrated that NSC745885 exhibits anti-oral cancer activity through the induction of apoptosis in cancer cells and in tumor-bearing mice, and this treatment did not induce marked toxicity in experimental mice. This compound also exhibits a comparable anti-tumor efficiency and a higher safety in experimental mice when compared to doxorubicin.

**Conclusions:**

The data of this study provide evidence for NSC745885 as a potential novel therapeutic drug for the treatment of human OSCC.

## Introduction

Among oral squamous cell carcinomas (OSCCs), the greatest majority of malignancies are head and neck squamous cell carcinomas (HNSCCs). OSCC is the sixth most prevalent cancer worldwide [1], and the third most common cancer in developing countries [2]–[4]. Traditional therapies for OSCC include surgery, radiotherapy, and chemotherapy. However, the remedial effect of such therapies on end-stage oral cancer is uniformly poor. In addition, even after successful tumor resection, approximately 20% of patients may have recurrence of tumors at other site [5]. Therefore, developing novel therapeutic strategies for malignant oral tumors is an urgent issue.

Anthracyclines and their anthraquinone derivatives exhibit cell growth inhibitory effects and have been used as anti-cancer drugs for more than 30 years [6]. Daunorubicin and doxorubicin, two anthracycline antibotics, are frequently used for the treatment of acute myeloid leukaemia as well as diverse solid tumors [7]. Previous studies have reported that these drugs induce apoptosis in tumor cells [8], [9]. The cytostatic and cytotoxic actions of these drugs have been observed through the inhibition of topoisomerase II and, subsequently, the initiation of DNA damage [10]. Oxidative damage has been considered a critical mechanism in the anti-tumor activity of anthracycline [11]. Anthracycline antibiotics also demonstrate the ability to reduce telomerase activity and hTERT expression. Doxorubicin treatment induces senescence in the breast tumor cell line MCF-7 cell through increased activity of p53, and reduces the activity of telomerase activity in this cancer cell [12]. A previous study reported that telomerase activity and hTERT mRNA expression were inhibited by doxorubicin treatment in parent gastric carcinoma cell lines but not in doxorubicin-resistant cell lines [13]. The results of these studies suggested an effect of anthracyclines in the inhibition of telomerase activity, and this effect contributed to the anti-tumor activity of these drugs.

Although anthracycline antibiotics have been used as anti-cancer drugs for many years, their cardiotoxicity raises a clinical therapy concern [14]. The features of anthracycline-induced cardiomyopathy in the biopsies of treated patients include myofibrils loss, sarcoplasmic reticulum dilation, cytoplasmic vacuolization, an increased number of lysosomes, and swelling of mitochondria in cardiomyocytes [15]. Previous studies have demonstrated that the development of cardiomyopathy in doxorubicin treatment is associated with the administered dose [16]. This side effect typically occurs within 1 year; however, it may also occur several years later during drug administration. Furthermore, anthracyclines can also lead to infrequent (less than 1%) acute cardiotoxicity (generally occuring within 1 week) [17]. Therefore, the development of a new drug exerting an efficient anti tumor effect, but exhibiting a minor side effect such as cardiotoxicity, is continues to be sought cancer therapy.

NSC745885 is a novel compound developed in 2009. This compound was synthesized from 1,2-diaminoanthraquinone, which subsequently reacts with thionyl chloride and triethylamine [18]. A previous study found that NSC745885 exhibits an anti-cancer effect when the 60 cell line primary screen of the National Cancer Institute (NCI) was used. The results indicated that leukemia, melanoma, and ovarian cancer lines were highly sensitive to NSC745885. Non small cell lung cancer, colon cancer, neuroblastoma, prostate cancer, and breast cancer cell lines are also sensitive to this compound. Furthermore, this previous study also reported that NSC745885 is effective in suppressing cell growth in various cancer cell lines, which is partially achieved by the inhibitory ability in the telomerase activity on these cells [18]. However, the *in vitro* anti-cancer potential and the *in vivo* activity of this novel compound in oral cancer has not been explored.

In this study, we investigated the anti-cancer potential of NSC745885 in oral squamous carcinoma cell lines and in an *in vivo* oral cancer xenograft mouse model. Furthermore, we also evaluated the toxicity of NSC745885 in other organs of treated mice. Our results demonstrated that this novel compound induced apoptosis of oral cancer cells either in the *in vitro* cell culture or in the *in vivo* xenograft tumor mouse model. In addition, we also found that *in vivo* treatment of NSC745885 elicited no cytotoxicity in the spleen, lung, liver or heart of the experimental animals. The finding of our study suggested that NSC745885 could be used as an effective drug for oral cancer therapy, and this treatment may exhibit a higher level of safety than traditional anthracycline antibiotics do.

## Materials and Methods

### Cells and Chemicals

SAS was obtained from a poorly differentiated human squamous cell carcinoma [19]. This cell line was provided by Dr. Jeng-Fan Lo of the Institute of Oral Biology, Department of Dentistry, National Yang-Ming University, Taiwan [20]. OECM-1 was obtained from gingival epidermoid carcinoma of a Taiwanese patient. SCC4 and SCC25 were obtained from tongue squamous cancer cells. All cell lines were maintained in an RPMI 1640 medium supplemented with 10% fetal bovine serum, 2 mM of L-glutamine, 25 mM of HEPES, and 1% penicillin/streptomycin. MRC-5 cell is a normal human fetal lung fibroblast cell line (ATCC No: CCL-171) maintained in an Minimum Essential Medium (MEM) supplemented with 10% fetal bovine serum, 2 mM of L-glutamine, 25 mM of HEPES, and 1% penicillin/streptomycin. NSC745885 [18] was provided by Dr. Hsu-Shan Huang of the Department of Pharmacy, National Defense Medical Center, Taiwan.

### Growth Inhibition Assay

Cells in the logarithmic growth phase were cultured at a density of 5000 cells/well in 96-well plates. The cells were exposed to various concentrations of NSC745885 for 24, 48, or 72 hours. A 3-(4,5-dimethylthiazol-2yl)-2,5-diphenyltetrazolium bromide (MTT) assay (Sigma, St Louis, MO, USA) was used to evaluate the effect of NSC745885 on cell growth, as described previously. The IC_50_ value resulted from 50% inhibition of cell growth, which was graphically calculated as a comparison with control growth.

### Annexin V staining

Cells were washed with PBS and resuspended in 1 X Binding Buffer (BD Pharmingen, San Diego, CA, USA). Resuspended cells were stained with FITC-conjugated Annexin V (BD Pharmingen, San Diego, CA, USA) and the percentage of Annexin V-positive cells was analyzed by flow cytometry.

### Quantitative Real-Time PCR

Total RNA was isolated using the Trizol method (Invitrogen, Carlsbad, CA, USA) and the homogenization of the cancer cells in Trizol lysis buffer was followed by chloroform extraction (Life Technologies). For cDNA synthesis, 5 µg of total RNA was reverse transcribed at 50°C for 60 minutes by using 200 units of Superscript III reverse transcriptase (Invitrogen, Carlsbad, CA, USA). Primer pairs for caspase-3 (sense 5′-CTG GAC TGT GGC ATT GAG AC-3′; antisense 5′-ACA AAG CGA CTG GAT GAA CC-3′), XIAP (sense 5′-GAC AGT ATG CAA GAT GAG TCA AGT CA-3′; antisense 5′-GCA AAG CTT CTC CTC TTG CAG-3′), and GAPDH (sense 5′-GGA AGG TGA AGG TCG GAG TCA-3′; antisense 5′-GTC ATT GAT GGC AAC AAT ATC CAC T-3′) were used for gene amplification. The SYBR Green/ROX qPCR master mix kit (Fermentas, Glen Burnie, Maryland, USA) was used for all reactions with a real-time polymerase chain reaction (PCR). In brief, PCR was performed as follows: one cycle at 50°C for 2 minutes, one cycle of 95°C for 10 minutes, followed by 40 cycles of 15 seconds each at 95°C, and 1 minute at 60°C. Data were collected in triplicate and normalized with GAPDH expression.

### Protein Extraction and Western Blot Analysis

Protein was extracted from a cultured cell (SAS) lysed in a cell lysis containing protease inhibitors (50 mmol/L of Tris (pH 7.5), 30 mmol/L of MgSO_4_, 8 mmol/L of EDTA, 2 mmol/L of DTT, and 2% Triton X-100). Cell lysates were clarified by centrifugation at 13000 rpm, at 4°C for 15 minutes. Protein concentrations of cell lysates were measured using a BCA assay (Thermo Scientific, Rockford, IL, USA). Western blot analysis was performed using 50 µg protein extracts from control cancer cells, and cells treated with NSC745885 were loaded and separated using 8% sodium dodecyl sulfate polyacrylamide gel electrophoresis. After electrophoresis, the proteins were electrotransferred onto a polyvinyldifluoride (PVDF) membrane (Millipore, Billerica, MA, USA), which was then blocked with a blocking solution containing 5% non-fat powdered milk in PBS plus 0.2% Tween 20 (PBST) for 1 hour at room temperature. After the blocking was completed, the membrane was washed 3 times with PBST. The PVDF membrane was blotted with the following primary antibodies in PBS: anti-XIAP; caspase-3 (Cell Signaling Technology, Danvers, MA, USA); and anti-actin (Sigma, St Louis, MO, USA). After 2 hours at room temperature, the membrane was again washed in PBST. The PVDF membrane was then incubated with peroxidase-linked anti-mouse or anti-rabbit IgG antibodies for 1 hour, developed using an enhanced chemiluminescence detection kit (Millipore, Billerica, MA, USA), and analyzed with a Las-3000 imaging system (Fujifilm, Tokyo, Japan).

### Xenograft Tumor Mouse Model

Eight-week-old NOD/SCID (NOD.CB17 *Prkdc* ^scid^/J, National Laboratory Animal Center, Taiwan) mice were maintained in micro-isolators under specific pathogen-free conditions and fed sterile food and chlorinated sterile water. Eighteen mice were divided into 2 groups, and each group of mice was subcutaneously injected with 3×10^6^ SAS. Nine mice in each group were further treated with NSC745885 or doxorubicin (2.0 mg/kg BW/d/i.p.), and 9 mice in each group were injected daily with a vehicle control. NSC745885 was first injected in each group of mice on day 3, before any tumor was palpated and continually administrated until day 10 in SAS-bearing mice. The size of the transplanted tumors was measured on every third day by using gauged calipers, and the tumor volume was calculated using the following formula: volume (V)  =  1/2× (length × width^2^). At the end of treatment, the mice were euthanized, and the tumors were removed, weighed, and photographed.

### Ethics statement

All animal experiments were conducted in accordance with institutional guidelines and were approved by NDMC's Institutional Animal Care and Use Committee. All efforts were made to minimize the suffering of experimental animals (approval number: IACUC-11-044).

### Immunohistochemical Staining

Immunohistochemical staining was conducted using the avidin-biotin method described as follows. Tissue sections were dewaxed in xylene and rehydrated in alcohol. Antigen retrieval was conducted through incubation in 0.01 mol/L of citrate buffer (pH 6.0) at 95°C for 40 minutes in a water bath. The endogenous peroxidase was blocked with 0.3% hydrogen peroxide for 30 minutes. The sections were then incubated with 5% normal horse serum in PBS for 30 minutes at room temperature to block a nonspecific antibody reaction. After washing with TBS and 0.1% Tween 20, the slides were incubated overnight at 4°C with anti-human caspase-3 and an XIAP antibody (DAKO, Osaka, Japan). After being rinsed in TBS and 0.1% Tween 20, the tissue sections were incubated for 40 minutes at room temperature with biotinylated anti-mouse IgG followed by an avidin-biotin-peroxidase complex (Vecstatin Elite ABC kit, Vector Laboratories, Inc., Burlingame, CA) for 40 minutes. Subsequently, the tissue sections were stained with 0.003% 3,3-diaminobenzide tetrahydrochloride and 0.005% hydrogen peroxide in 0.05 mol/L of Tris-HCl (pH 7.2), counterstained with Mayer's hematoxylin, dehydrated, and mounted.

### Evaluation of Immunohistochemical Staining

An immunohistochemical procedure without adding the primary antibody was used as a negative control in each case. The intensity of caspase-3 and XIAP immunoreactivity of the tumor cells were scored into 3 groups, and standardized according to the staining level as a positive internal control on a scale of 0 (no staining), 1 (weak staining), 2 (moderate staining), and 3 (strongest intensity). The distribution of caspase-3 and XIAP labeling was also measured as according to the percentage of positively stained tumor cells (from 0 to 100) in the total tumor volume in each section. To evaluate the distribution areas of caspase-3 and XIAP expressions more easily and effectively, 50% was considered the cutoff point. To compare the control and NSC745885-treated group, the percentage of caspase-3 and XIAP-positive cells at each intensity was multiplied by the corresponding intensity (from 0 to 3) to obtain an immunostaining score ranging from 0 to 300.

## Statistical Analysis

The Statistical Package for the Social Sciences (SPSS Version 10.0 for Microsoft Windows, Chicago, IL, USA) was used to complete the analysis of the collected data. A *t*-test and one-way analysis of variance (ANOVA) with Scheffe's post-hoc test were used to determine whether any significant relationships existed among the quantitative results. Values of *P* < 0.05 were considered significant.

## Results

### Growth Inhibitory Effect of NSC745885 on Oral Cancer Cell Lines

**Figure 1:**
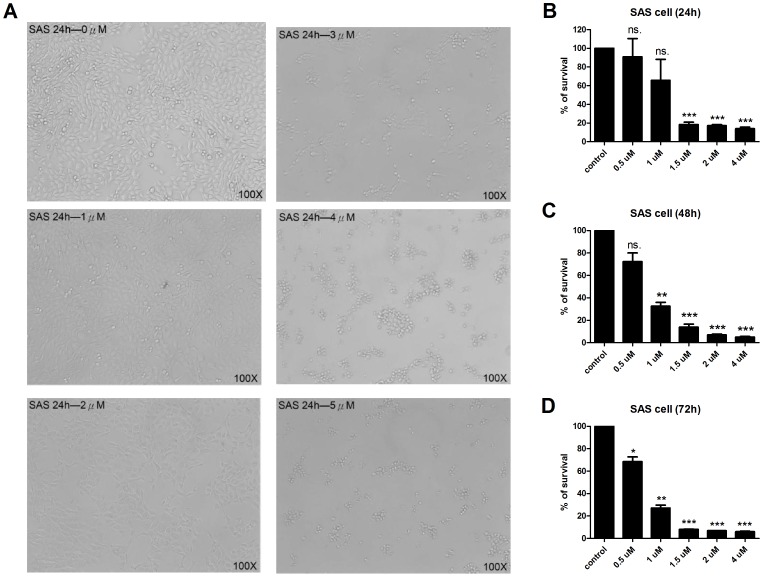
NSC745885 inhibits SAS cell growth in vitro. (A) Morphological changes in SAS cells. Cells were treated with indicated dosages of NSC745885 for 24 hours. Dosages higher than 3 µΜ caused cell death in SAS cells. The survival of NSC745885 treated SAS cells was assessed by MTT assay at various times and dosages. (B) At 24 hours post treatment, the survival of SAS cells showed a significant difference at the dosages higher than 1 µM. (C) At 48 hours, treatment at 1 µM or higher than that dosages of NSC745885 exhibited a significant anti-cancer effect. (D) After 72 hours of treatment, the IC50 of NSC745885 was 0.85 µM in SAS cells. Survival proportion (%) indicated the relative value compared with vehicle control groups in SAS (n  =  3; *, P<0.05; **, P<0.01; ***, P<0.001; ns., non-significant).

**Figure 2:**
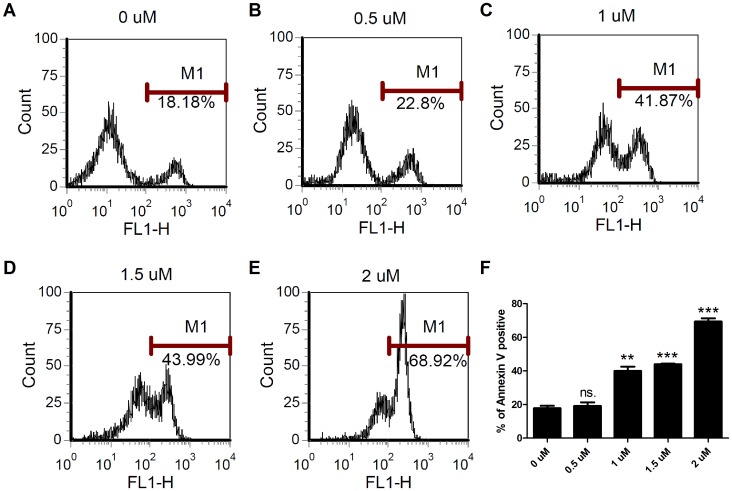
NSC745885 induces early apoptosis in SAS cells. SAS cells were treated with (A to E) indicated doses of NSC745885 for 24 hours. Harvested cells were stained with FITC-conjugated annexin V and analyzed by flow cytometry. (F) The percentages of annexin V positive cells under NSC745885 treatment were compared with vehicle controls (0 µM) (n  =  3; **, P<0.01; ***, P<0.001; ns., non-significant).

To examine the influence of NSC745885 treatment on oral squamous cell carcinoma, we treated the SAS oral cancer cells with various concentrations of NSC745885 for 24 hours and observed their morphological changes by using a phase-contrast microscope. The treatment of NSC745885 at the concentrations of 3 µM to 5 µM significantly decreased the densities of cultured cells when compared with untreated cells (Fig. 1A). To evaluate the growth inhibitory effect of NSC745885, the SAS cells were conducted with various doses of NSC745885, and the numbers of the surviving cells were measured at different time points and compared with those of untreated cells by MTT assay. The numbers of surviving SAS cells were significantly reduced following the NSC745885 treatment in time- and dose-dependent manners (Fig. 1B–1D). The IC_50_ of NSC745885 was 0.85 µM on the SAS cells after 72 hours of treatment (Fig. 1D). These results indicated that NSC745885 demonstrated a growth inhibitory or death-promoting effect on the SAS cells. To detect apoptotic SAS cells, we performed annexin V staining to evaluate the proportions of annexin V positive under various doses of NSC745885 treatment at 24 hours. The percentages of annexin V positive cells were increased in a dose-dependent manner (Fig. 2A–2E). There are significant differences when treatment dosage was higher than 0.5 µM (Fig. 2F). This result indicated that NSC745885 treatment induced early apoptosis in SAS cell.

**Figure 3:**
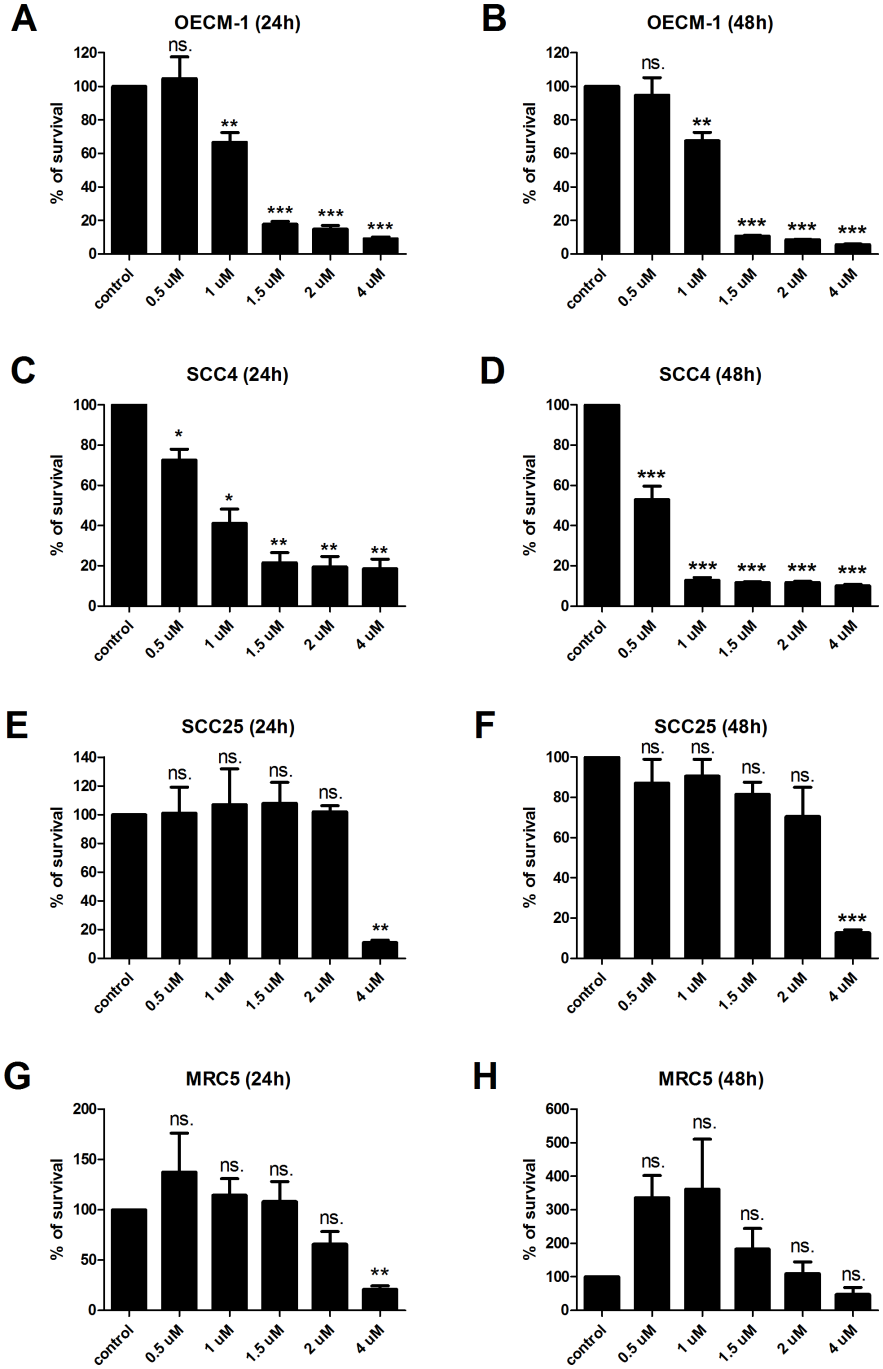
NSC745885 treatment exhibits a cancer cell specific cytotoxicity. Oral cancer cells (OECM-1, SCC4, and SCC25) and normal lung fibroblast MRC-5 cell were treated with NSC745885 for 24 or 48 hours with indicated concentrations. The viability of these cell lines were assessed by MTT assay.The results indicated that low micromolar concentrations of NSC745885 induced (A) SAS cell death but did not affect the growth of (G and H) MRC-5 cells (n  =  3; *, P<0.05; **, P<0.01; ***, P<0.001; ns., non-significant).

Furthermore, we also examined the growth inhibitory effect in other oral cancer cell lines, including OECM-1, SCC25, and SCC4. We found that NSC745885 also exhibited a significant growth inhibitory effect in OECM-1 cells at the dosages of 1 µM or higher at either 24 or 48 hours after drug treatment (Fig. 3A and 3B). This compound even exhibited a higher inhibitory efficacy in SCC4 cells (Fig. 3C and 3D). By contrast, there are significant differences only at 4 µM on SCC25 cells at either 24 or 48 hours (Fig. 3E and 3F). In order to investigate the influence of NSC745885 in normal tissues, we also evaluated the cytotoxic effect of this drug in MRC-5 cells (a normal human fetal lung fibroblast cell line) [21]. Our results found that only high concentrations of NSC745885 (4 µM) significantly affect the growth of MRC-5 cells (Fig. 3G and 3H), indicating that NSC745885 exhibits a specificity for cancerous cells.

### NSC745885 Treatment Induces Apoptosis of SAS Cells

**Figure 4:**
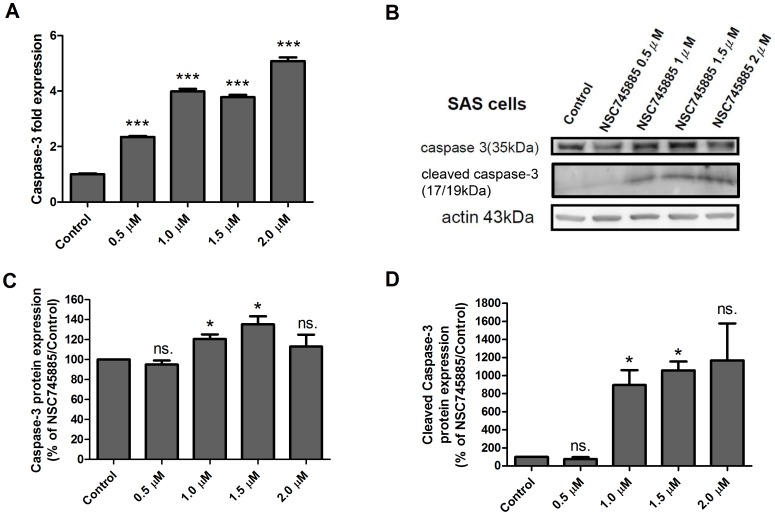
NSC745885 treatment enhanced the expression and activation of caspase-3 in SAS cells. (A) The relative RNA expression level of caspase-3 in SAS cell treated with indicated dosages of NSC745885 for 24 hours was evaluated using real-time RT-PCR. The expression of caspase-3 significantly increased with NSC745885 treatment in a dosage-dependent manner. (B) The caspase-3 and cleaved caspase-3 protein levels in the SAS cell treated with NSC745885 for 48 hours was quantified using western blot. (C) The results of the western blot in caspase-3 were evaluated in 3 independent experiments and normalized with β-actin (*, P < 0.05; **, P < 0.01; ***, P < 0.001; ns., non-significant). The protein level of caspase-3 was highest in 1.5 µΜ of NSC745885 treatment. (D) The results of the western blot in cleaved caspase-3 were evaluated in 3 independent experiments (*, P < 0.05; ns., non-significant).

To examine the effect of NSC745885 on the induction of apoptosis in SAS cells, the cells were treated with various doses of NSC745885 for 24 hours, and the mRNA expression levels of caspase-3 were measured using real-time RT-PCR. Our result indicated that the expression of caspase-3 was significantly increased with NSC745885 treatment in a dose-dependent manner (Fig. 4A). Protein levels of caspase-3 and cleaved caspase-3 in the SAS cells were also determined using western blot. There were significant increases in caspase-3 and cleaved caspase-3 under treatment with concentrations higher than 1 µM (Fig. 4B). The relative protein levels among various doses were compared using a density meter, displaying consistent results with direct observation in western blot (Fig. 4C and 4D). These data indicated that NSC745885 treatment induced the apoptosis of the SAS cells.

### NSC745885 Decreases the RNA and the Protein Expression of XIAP in SAS Cells

**Figure 5:**
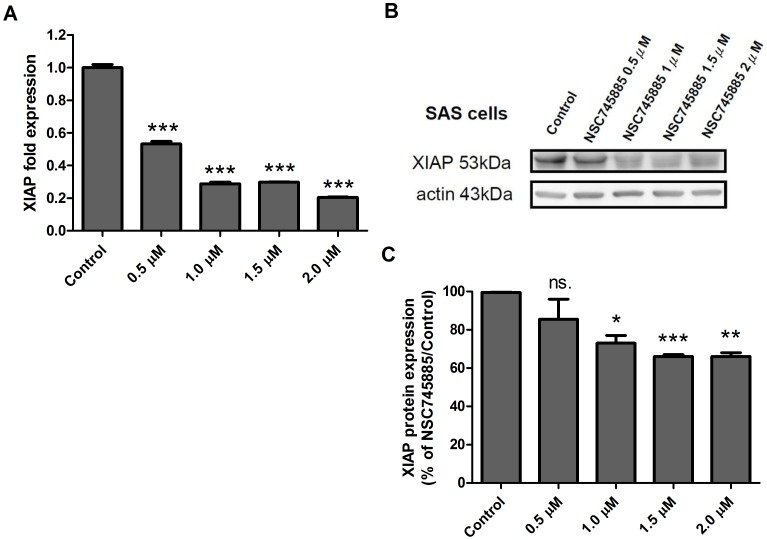
NSC745885 treatment decreased the RNA and protein levels of XIAP in SAS cells. The relative RNA expression level of XIAP in SAS cells treated with indicated dosages of NSC745885 was evaluated at 24 hours using real-time RT-PCR. The expression of XIAP significantly decreased with NSC745885 treatment in a dose-dependent manner. (B) The expression of XIAP in SAS cells treated with NSC745885 was quantified using western blot at 48 hours of treatment. (C) The western blot results were evaluated in 3 independent experiments and normalized with β-actin (*, P < 0.05; **, P < 0.01; ***, P < 0.001). The protein level of XIAP decreased in a dose-dependent manner.

The X-linked inhibitor of apoptosis protein (XIAP) has been identified as an anti-apoptotic protein in mammalian cells [22]. It plays a critical role in the inhibition of apoptosis in both death receptor-mediated and mitochondria-mediated pathways [23], [24]. A recent study further demonstrated that XIAP is a predictor of chemotherapy response and prognosis for advanced head and neck cancer patients [25]. Therefore, we also examine the effect of NSC745885 on the XIAP gene and protein expression in oral cancer cells, we treated the SAS cancer cells with different concentrations (0 µM, 0.5 µM, 1.0 µM, 1.5 µM, and 2.0 µM) for 24 hours, and then determined their change in gene expression by using the Q-PCR. Compared with the control cells, the expression XIAP gene significantly decreased with NSC745885 treatment in a dose-dependent manner (Fig. 5A). We also treated the SAS cancer cells with different concentrations (0 µM, 0.5 µM, 1.0 µM, 1.5 µM, and 2.0 µM) for 48 hours, and then determined their protein expression changes by using western blot. A significant decrease in XIAP was observed under treatment with concentrations higher than 1 µM (Fig. 5B). The relative protein levels among various doses were also compared using a density meter, displaying consistent results with direct observation in western blot (Fig. 5C). These results indicated that the NSC745885 exhibited an apoptosis-promoting effect in the SAS cells.

### NSC745885 Inhibits SAS Cell Growth *in vivo*

**Figure 6:**
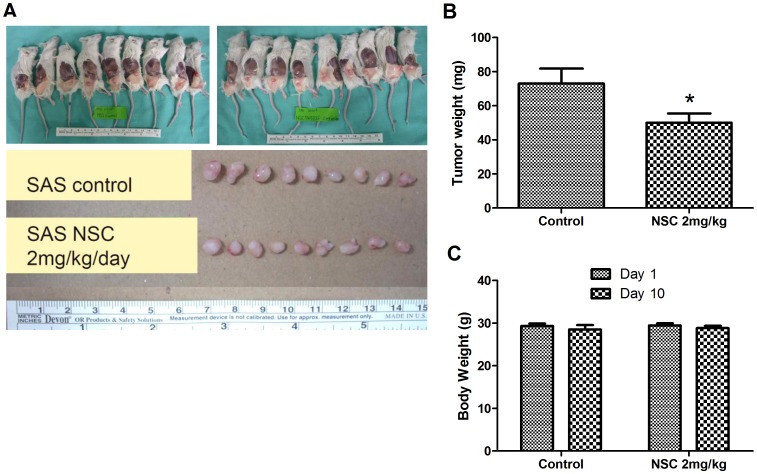
NSC745885 treatment inhibited SAS cell growth in vivo. (A) The cancer cell implanted NOD/SCID mice were administered with NSC745885 (2 mg/kg/d). Engrafted tumors were isolated on Day 10 after drug administration. NSC745885 treatment significantly reduced tumor size when compared with the vehicle control. (B) The average tumor weight was compared between the NSC745885-treated and PBS-treated tumor-bearing mice on Day 10 (n  =  9; *, P < 0.01). (C) The body weight of the experimental mice did not significantly decrease with NSC745885 treatment compared with the PBS treated controls.

To evaluate the anti-tumor potential of NSC745885 on oral cancer *in vivo*, we conducted this treatment in a xenograft tumor-bearing mouse model. The SAS cells were inoculated into NOD/SCID mice and NSC745885 was injected on day 3 in each group of mice, before any tumor was palpated, and administrated daily until day 10 in bearing mice. The tumor size was reduced in the treated mice compared with the tumor-bearing control mice that were treated with the vehicle alone (Fig. 6A). The SAS xenografts reduced in weight by 23 ± 10.39% with the NSC745885 treatment (Fig. 6B). The body weights of the control and NSC745885-treated mice were also assessed on day 1 and 10 after drug administration. No significant differences existed in the body weight of the control or NSC745885-treated mice (Fig. 6C). These results indicated that the NSC745885 treatment at 2 mg/kg exhibited an anti-oral cancer effect, and this treatment did not lead to marked toxicity in experimental mice.

### NSC745885 Induce Apoptosis in Xenografted Tumor Cells

**Figure 7:**
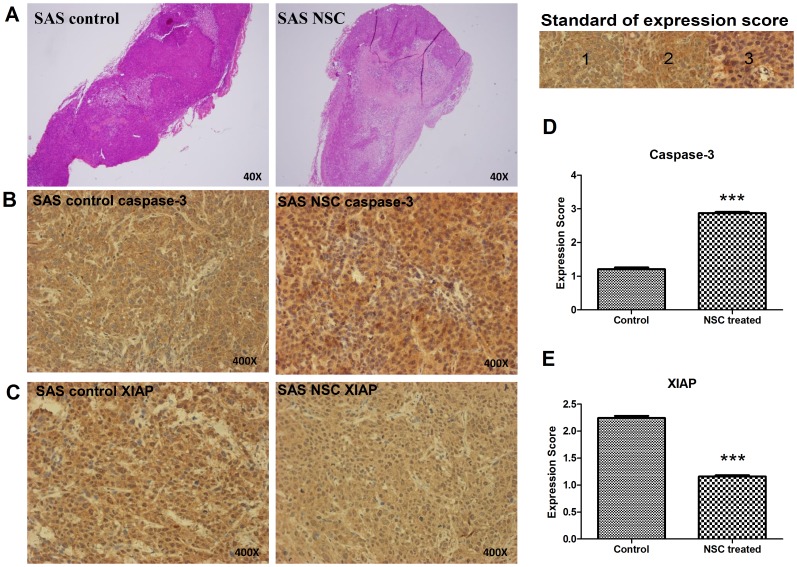
NSC745885 treatment induced apoptosis in xenografted tumors. (A) The histological changes in tumor grafts were assessed using HE staining. Comparing the PBS control with the NSC745885-treated groups revealed a marked tumor necrosis in the NSC745885-treated group. (B) The expression level of caspase-3 in the isolated tumor grafts were evaluated using immunohistochemical staining. (C) The expression level of XIAP in isolated tumor grafts were also evaluated using immuno-histochemical staining. The expression scores for caspase-3 (D) and XIAP (E) were quantified, revealing an increase in the expression of caspase-3 with NSC745885 treatment (P < 0.05). By contrast, the expression of XIAP in tumor grafts decreased with this treatment (P < 0.05).

To observe directly the apoptotic effect of NSC745885 on tumor cells *in vivo*, we performed immunohistochemical staining to evaluate the status of the apoptotic protein caspase-3 in xenografts. After 10 days of transplantation, the xenografts were removed, measured for size, and examined with HE and immunohistochemical staining. The tumors from the NSC745885-treated mice exhibited tissue damage in tumor part (Fig. 7A) and a markedly higher count of apoptotic cells (caspase-3 positive cell) compared with the control tumors (Fig. 7B). We also evaluated the expression of XIAP in the xenografts. The result revealed that the NSC745885-treated mice exhibited a markedly lower count of anti-apoptotic cells (XIAP positive cell) compared with the control tumors (Fig. 7C). The expression scores of the caspase-3 positive cells were 1.21 ± 0.04 and 2.87 ± 0.03 in the control group and NSC745885-treated group, respectively (Fig. 7D). The expression scores of the XIAP positive cells were 2.24 ± 0.03 and 1.16 ± 0.02 in the control group and NSC745885-treated group, respectively (Fig. 7E). Consequently, our results demonstrated that NSC745885 reduces the proliferation of SAS cancer cells, and exerted anti-tumor effect *in vivo* through the induction of apoptosis.

### Effects of NSC745885 on Body Weight in Mice

**Figure 8:**
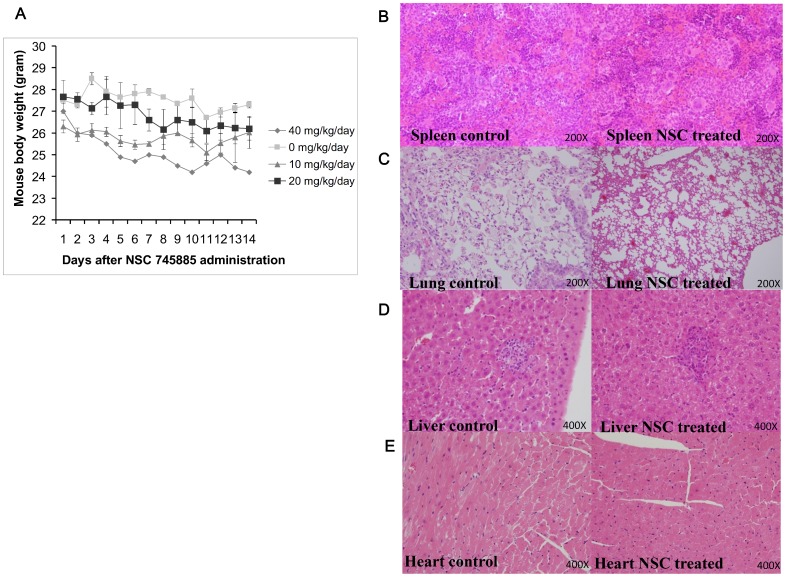
The effects of NSC745885 treatment in the experimental mice. (A) NOD/SCID mice were treated with PBS or indicated daily dosages of NSC745885, and the body weights of these mice were measured. The influence of NSC745885 treatment on the organs of the experimental mice was assessed using a histological assay and HE staining on Day 14. No obvious differences existed in the (B) spleen, (C) lung, (D) liver, or (E) heart of PBS-treated and NSC745885-treated mice.

To determine whether NSC745885 treatment causes body weight loss, we monitored the alteration of body weight in the experimental mice. A significant cytotoxic effect of NSC745885 was revealed with the daily administration of NOD/SCID at a dosage of 40 mg/kg/d (Fig. 8A). The body weight of mice treated with the higher dosage was significantly decreased, and all of the mice died by day 14. In this study, we found that the maximum tolerated dose of NSC745885 in mice was 40 mg/kg/d. To determine whether NSC745885 treatment created cytotoxicity in the organs of the experimental mice, the spleens, lungs, livers, and hearts were harvested from the PBS-treated and NSC745885-treated mice on day 14 and were assessed using a histological assay. No obvious differences existed in the organs of PBS-treated or NSC745885-treated mice (Figs. 8B–8E). This result indicated that the daily treatment of NSC745885 at 2.0 mg/kg/d i.p. elicited no marked adverse effects in the mice.

### Comparisons of the Safety and the Anti-tumor Efficiency between NSC745885 and Doxorubicin

**Figure 9:**
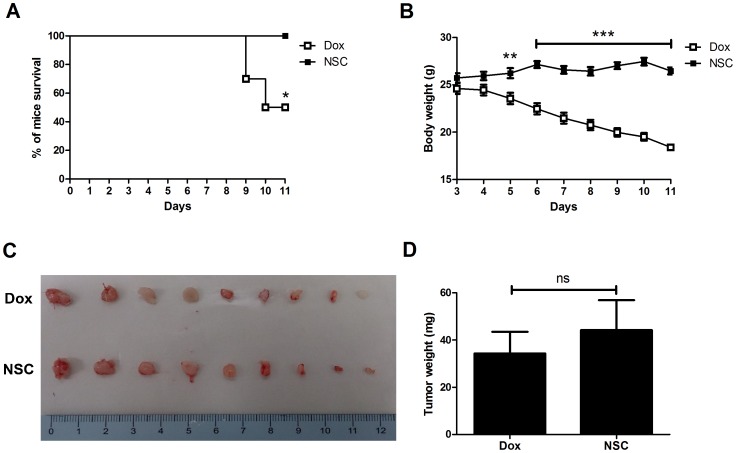
Comparisons of the safety and the anti-tumor efficiency between NSC745885 and doxorubicin. The cancer cell implanted NOD/SCID mice were administered with NSC745885 (NSC, 2 mg/kg/d) or doxorubicin (Dox, 2 mg/kg/d) on day 3. (A) The survival rates between NSC745885 and doxorubicin treated mice were assessed. There are no experimental mice were dead under NSC745885 treatment by day 11. By contrast, 50% of doxorubicin treated mice were dead (n = 10). (B) The body weights of doxorubicin treated mice were significant decreased when compared to NSC745885-treated group from day 3 to day 11 after the beginning of drug treatment. (C) SAS cells were inoculated into NOD/SCID mcie, and the treatments of doxorubicin or NSC745885 were started at day 3 after cell inoculation. Engrafted tumors were isolated on day 11 after SAS cell inoculation (n = 9). (D) There are no significant differences between NSC745885-treated and doxorubicin-treated groups (n = 9).

To compare the safety of NSC745885 with doxorubicin, we treated NOD/SCID mice with these two compounds at 2 mg/kg/d i.p. for 10 days. The survival and body weight of experimental mice were monitored daily. Our results showed that 50% of doxorubicin-treated mice were dead at day 11 (Fig. 9A). By contrast, all NSC745885-treated mice were surviving at day 11 (Fig. 9A). There are significant decreased in the body weights of doxorubicin-treated mice when compared to NSC745885-treated groups (Fig. 9B). These results indicated that NSC745885 exhibited a higher safety than doxorubicin. Furthermore, we also compared the anti-tumor efficiency between these two drugs by measuring the tumor weights in xenograft tumor-bearing model. NOD/SCID mice were inoculated with SAS cells and treated with 2 mg/kg/d i.p. of doxorubicin or NSC745885 from day 3 to day 10 post cell inoculation. Tumors were harvested at day 11 (Fig. 9C, n = 9) and tumor weights were measured. There are no significant difference between doxorubicin-treated and NSC745885-treated groups (Fig. 9D, n = 9), indicating a comparable anti-tumor efficiency between these two drugs.

**Figure 10:**
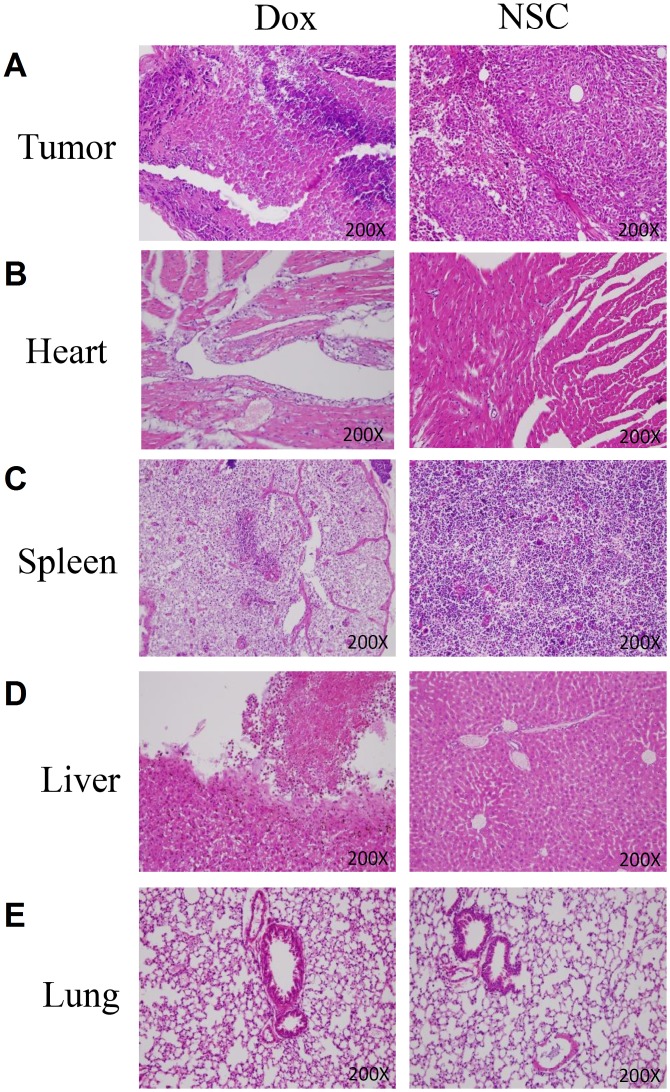
Pathohistological analysis in doxorubicin-treated and NSC745885-treated mouse. The histological changes in (A) tumors, (B) hearts, (C) spleens, (D) liver and (E) lungs which isolated from doxorubicin-treated and NSC745885-treated mouse were assessed by using HE staining.

In addition, we also isolated organs from doxorubicin-treated and NSC745885-treated mice and performed pathohistological analysis by HE staining. We found that there are multifocal tumor necrosis in either doxorubicin-treated and NSC745885-treated groups (Fig. 10A). Acute endomyocarditis with myocytes necrosis was presented in the heart of doxorubicin-treated mouse, but no significant change in NSC-treated mouse (Fig. 10B). In spleen, complete autolysis was shown in doxorubicin-treated group, and only focal autolysis in NSC-treated group (Fig. 10C). There are also focal hepatocytes necrosis in doxorubicin-treated mouse, but no significant change in NSC-treated mouse (Fig. 10D). There are no significant change in the lungs of doxorubicin- and NSC-treated mouse (Fig. 10E). These results further confirmed that NSC745885 exhibited a higher safety than doxorubicin, especially in the heart and liver of experimental mice.

## Discussion

Chemotherapy, which is based on cytostatic and cytotoxic agents targeting the cellular mechanisms involved in controlling of cell growth and division [26], is frequently used in conjunction with surgery. Anthracyclines antibiotics, such as daunorubicin and doxorubicin, have been used as anti-cancer drugs in clinical cancer therapy [6]. However, the cardiotoxicity of these drugs limits their clinical use [14]. Therefore, the development of novel drugs with higher efficiency and less cardiotoxicity than those of traditional anthracyclines for cancer is urgent. NSC745885 is a novel compound developed in 2009, and the anti-tumor effect of this compound in leukemia and certain solid tumor cell lines was tested in a previous study [18]. It also has been found to induce HeLa cell apoptosis via regulation of DNA damage [27]. However, the effect of this novel compound on OSCCs remains unclear. Furthermore, the *in vivo* anti-tumor effect of NSC745885 has not been evaluated in any animal model of cancers.

In this study, we first examined the anti-cancer potential of NSC745885 in OSCC cell lines. We found that NSC745885 induced cell death of these cell lines, and the expression of caspase-3 in SAS cells was significantly increased, and the expression of the anti-apoptotic protein XIAP was significantly reduced. In addition, the increased numbers of the caspase-3 signal were also observed in the xenograft tumor mass of mice treated with NSC745885. By contrast, the presence of XIAP in the xenograft tumor mass decreased with NSC745885 treatment. Because XIAP has been demonstrated to be a potential target for anti-cancer therapy [28], the effect of NSC745885 in the suppression of this critical regulator of apoptosis may demonstrate a therapeutic potential for treating oral cancer. However, the detail mechanism for the suppression of XIAP expression by NSC745885 remains unclear and could be further investigated in the future. Furthermore, our data also found that this compound caused lower cytotoxicity in normal human lung fibroblast cells when compared with that of cancer cells, suggesting a cancer cell specific effect of NSC745885 treatment. Our data first provided evidence for the death-promoting effect of NSC745885 through the induction of apoptosis in oral cancer cells.

A telomere is a unique chromosomal sequence containing a tandem GT-rich repeat that protects chromosomes from fusion or degradation [29]. The length of a telomere progressively shortens with cell division and results in cellular senescence. However, germ cells and immortal cells, such as cancer cells, possess the ability to maintain the length of a telomere [29]. Telomerase is a ribonucleoprotein enzyme complex that exhibits a reverse transcriptase activity to elongate telomeric DNA, and has been found to play a critical role in the development of cellular immortality and oncogenesis [29]. Previous studies have indicated that anthracyclines treatment inhibits telomerase activity and hTERT mRNA expression in cancer cells, and these effects may contribute to the anti-tumor activity of these drugs [12], [13]. However, Huang *et al*. demonstrated that NSC745885 showed no telomerase inhibitory activity or a specific repressive effect in the hTERT gene [18]. Therefore, the mechanism of NSC745885 in tumor suppressive activity is less likely through the inhibition of telomerase activity or hTERT expression.

The cardiotoxicity of anthracycline antibiotics is a major concern in their clinical application [14]. However, the cytotoxicity of this novel compound in cardiomyocyte has not been evaluated in previous studies [18]. In this study, we evaluated the toxicity of NSC745885 in mice by measuring changes in their body weight. We also evaluated the influence of NSC745885 on the spleen, lung, liver, and heart of treated mice by using a histo-pathological analysis. In the dosage of 2 mg/kg/d, no marked histopathological changes were observed, suggesting a low toxicity to experimental animals. Previous studies have demonstrated that doxorubicin treatment in the dose of 2 mg/kg weekly for 3 weeks or 2.5 mg/kg weekly for 5 weeks induces heart failure in Sprague-Dawley rats [30], [31]. Other study also documented that treatment of doxorubicin in the dosage of 2 mg/kg/week for 12 weeks induces cardiomyopathy in mice [32]. In our study, treatment with NSC745885 at equal dose but higher intensity did not lead to marked cardiotoxicity in experimental mice. Our data also indicated that this compound leads to lower cytotoxicity in normal human lung fibroblast cells when compared with that of cancer cells. We also compared the anti-tumor efficiency and safety of NSC745885 to doxorubicin *in vivo*. Our data indicate that NSC745885 exhibited a comparable anti-tumor efficiency with doxorubicin. Furthermore, treatment of this compound did not lead to death and induced a lower cytotoxicity in heart and liver in experimental mice when compared to that of doxorubicin. These results suggest that this novel compound exhibits a lower toxicity to normal cells and a higher safety than doxorubicin to experimental mice.

In conclusion, this study demonstrate that the novel compound NSC745885 possesses anti-oral cancer activity through the induction of apoptosis in cancer cells, and this treatment did not leads to marked toxicity in experimental mice. These results provide evidence for NSC745885 as a potential therapeutic drug for treating human OSCC.
