# Correction: A Novel Compound NSC745885 Exerts an Anti-Tumor Effect on Tongue Cancer SAS Cells In Vitro and In Vivo

**DOI:** 10.1371/journal.pone.0113703

**Published:** 2014-11-11

**Authors:** The PLOS ONE Staff

There are errors in the Funding section. The correct funding information is as follows: This study was supported by research grants from the National Science Council, Taiwan, Republic of China (NSC101-2320-B-016-016 to G.-J. Lin, NSC102-2314-B-016-018-MY3 to Y.-W. Chen and NSC102-2314-B-016-032-MY2 to S.-H. Huang), Tri-Service General Hospital, Republic of China (Grant No. TSGH-C102-007-009-S06 and TSGH-C103-005-007-009-S06), Ministry of National Defense, Republic of China (103-M075 to G.-J. Lin and 103-M055 to Y.-W. Chen) and in part by the C.Y. Foundation for Advancement of Education, Science and Medicine. The funders had no role in study design, data collection and analysis, decision to publish, or preparation of the manuscript.

In the Author Contributions section, the initials YWC GJL are inadvertently repeated. Please refer to the correct Author Contributions here: Conceived and designed the experiments: YWC GJL. Performed the experiments: YWC SHH DYH. Analyzed the data: HSH YSS KHM. Contributed reagents/materials/analysis tools: HSH HKS. Wrote the paper: YWC GJL.
